# SARS-CoV-2 envelope protein topology in eukaryotic membranes

**DOI:** 10.1098/rsob.200209

**Published:** 2020-09-09

**Authors:** Gerard Duart, Mª Jesús García-Murria, Brayan Grau, José M. Acosta-Cáceres, Luis Martínez-Gil, Ismael Mingarro

**Affiliations:** Departament de Bioquímica i Biologia Molecular, Estructura de Recerca Interdisciplinar en Biotecnologia i Biomedicina (ERI BioTecMed), Universitat de València E-46100 Burjassot, Spain

**Keywords:** coronavirus, envelope protein, membrane insertion, SARS-CoV-2, topology

## Abstract

Coronavirus E protein is a small membrane protein found in the virus envelope. Different coronavirus E proteins share striking biochemical and functional similarities, but sequence conservation is limited. In this report, we studied the E protein topology from the new SARS-CoV-2 virus both in microsomal membranes and in mammalian cells. Experimental data reveal that E protein is a single-spanning membrane protein with the N-terminus being translocated across the membrane, while the C-terminus is exposed to the cytoplasmic side (Nt_lum_/Ct_cyt_). The defined membrane protein topology of SARS-CoV-2 E protein may provide a useful framework to understand its interaction with other viral and host components and contribute to establish the basis to tackle the pathogenesis of SARS-CoV-2.

## 1. Introduction

Coronavirus disease 2019 (COVID-19), an extremely infectious human disease caused by coronavirus SARS-CoV-2, has spread around the world at an unprecedented rate, causing a worldwide pandemic. While the number of confirmed cases continues to grow rapidly, the molecular mechanisms behind the biogenesis of viral proteins are not fully unravelled. The SARS-CoV-2 genome encodes up to 29 proteins, although some may not get expressed [1]. The viral RNA is packaged by the structural proteins to assemble viral particles at the ERGIC (ER-Golgi intermediate compartment). The four major structural proteins are the spike (S) surface glycoprotein, the membrane (M) matrix protein, the nucleocapsid (N) protein, and the envelope (E) protein. These conserved structural proteins are synthesized from sub-genomic RNAs (sgRNA) encoded close to the 3′ end of the viral genome [2].

**Figure 1.**
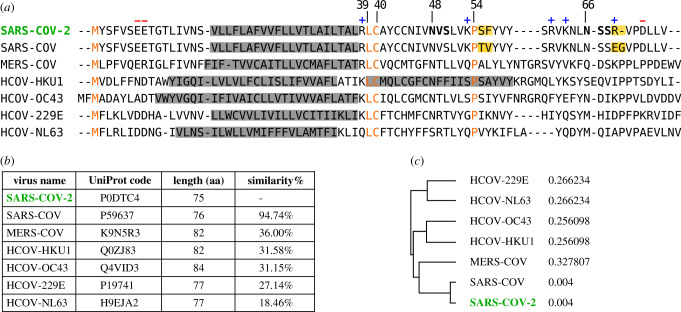
(*a*) Multi-alignment of amino acid sequences of the E protein of SARS-CoV-2 and the other six human coronavirus. SARS-CoV severe acute respiratory syndrome coronavirus (UniProt P59637), MERS-CoV Middle East respiratory syndrome coronavirus (UniProt K9N5R3), HCoV-HKU1 (UniProt Q0ZJ83), HCoC-OC43 (UniProt Q4VID3), HCoC-229E (UniProt P19741) and HCoV-NL63 (UniProt Q5SBN7). Predicted TM segments at UniProt are highlighted in a grey box. Native predicted glycosylation acceptor sites in SARS-CoV-2 are shown in bold and charged residues highlighted with + or – symbols on top. Conserved residues are shown in orange. Differences between SARS-CoV-2 and SARS-CoV are highlighted as yellow boxes. (*b*) Phylogenetic data and (*c*) tree obtained with Clustal Omega (EMBL-EBI) using the default parameters.

Among the four major structural proteins, the E protein is the smallest and has the lowest copy number of the membrane proteins found in the lipid envelope of mature virus particles (reviewed [3,4]). However, it is critical for pathogenesis of other human coronaviruses [5,6]. Interestingly, the sgRNA encoding E protein is one of the most abundantly expressed transcripts despite the protein having a low copy number in mature viruses [1]. It encodes a 75 residues long polypeptide with a predicted molecular weight of approximately 8 kDa. Two aliphatic amino acids (Leu and Val) constitute a substantial portion (36%, 27/75) of the E protein, which accounts for the high grand average of hydropathicity (GRAVY) index of the protein (1.128), as calculated using the ExPASy ProtParam tool (https://web.expasy.org/protparam/). Comparative sequence analysis of the E protein of SARS-CoV-2 and the other six known human coronaviruses do not reveal any large homologous/identical regions (figure 1), with only the initial methionine, Leu39, Cys40 and Pro54 being ubiquitously conserved. With regard to overall sequence similarity SARS-CoV-2 E protein has the highest similarity to SARS-CoV (94.74%) with only minor differences (figure 1*b*), followed by MERS-CoV (36.00%). Interestingly, sequence similarities are significantly lower for the other four human coronaviruses, which usually cause mild to moderate upper-respiratory tract illness typical for common cold, with the lowest similarity found for HCOV-NL63 (18.46%). These findings are consistent with the phylogenetic tree proposed based on the amino acid sequences of the human coronavirus E proteins using ClustalW (figure 1*c*).

## 2. Results and discussion

### 2.1. E protein topology prediction

Computer-assisted analysis of the SARS-CoV-2 E protein amino acid sequence using seven popular prediction methods showed that all membrane protein prediction algorithms except MEMSAT-SVM suggested the presence of one transmembrane (TM) segment located roughly around amino acids 12 to 39 (table 1), which is not predicted as a cleavable signal sequence according to SignalP-5.0 [7]. Regarding E protein topology, TMHMM and Phobius predicted an N-terminus cytosolic orientation, while MEMSAT-SVM, TMpred, HMMTop and TOPCONS predicted an N-terminus luminal orientation. These discrepancies found among the predictions from different algorithms motivated experimental approaches.

**Table 1.** Computer analysis of the SARS-CoV-2 E protein amino acid sequence topology. n.p., non-predicted.

algorithm	Nt	Ct	TMDs (start-end)
ΔG predictor	n.p.	n.p.	1 (17–39)
TMHMM	cytosol	lumen	1 (12–34)
MEMSAT-SVM	lumen	lumen	2 (10–39) (43–58)
TMpred	lumen	cytosol	1 (17–34)
HMMTop	lumen	cytosol	1 (11–35)
Phobius	cytosol	lumen	1 (12–37)
TOPCONS	lumen	cytosol	1 (16–36)

### 2.2. Insertion into microsomal membranes

**Figure 2.**
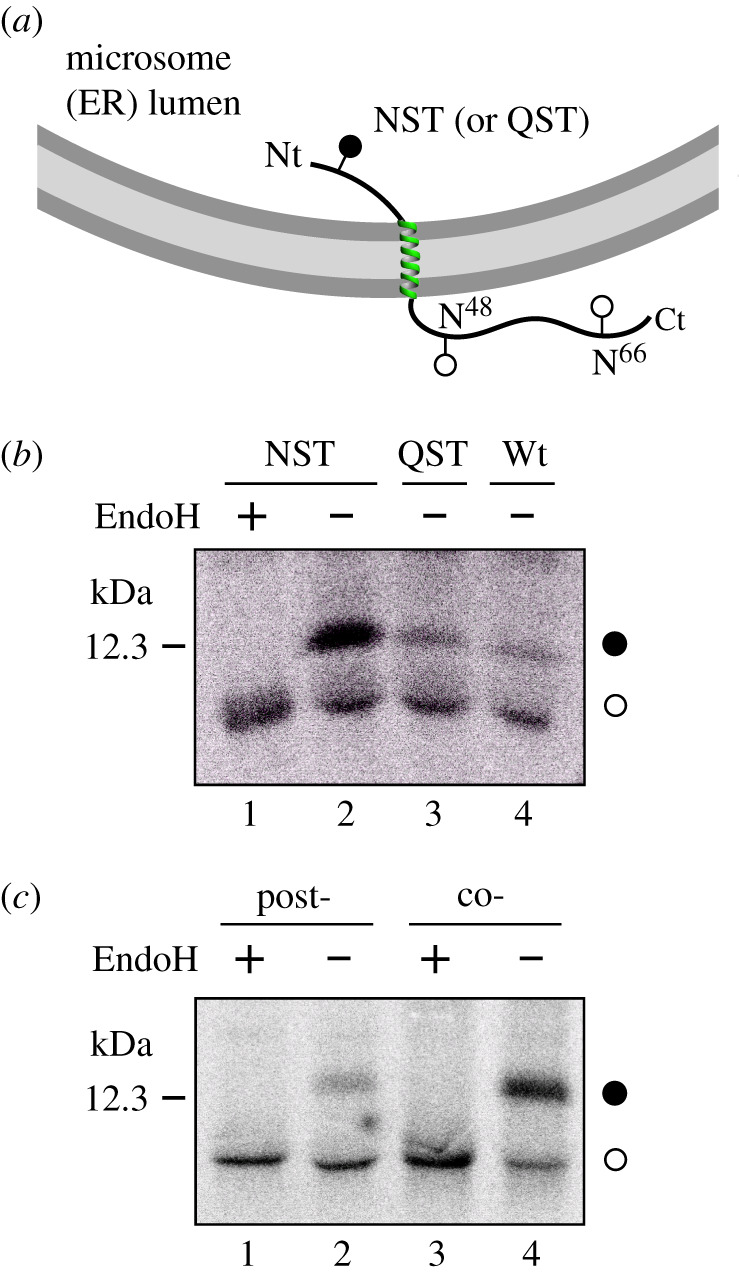
Translocon-mediated insertion of E protein variants into microsomal membranes. (*a*) Schematic representation of E protein constructs. Glycosylation acceptor Asn residues are indicated. (*b*) *In vitro* translation in the presence of microsomes of the different E protein constructs. Construct containing inserted asparagine and threonine residues at positions 3 and 5 (NST; lanes 1–2) or glutamine and threonine at positions 3 and 5 (lane 3), and wild-type variants (lane 4) were translated in the presence of microsomes. NST variant was split and half of the sample was Endo H treated (lane 1). Bands of non-glycosylated and glycosylated proteins are indicated by white and black dots, respectively. (*c*) E protein (harbouring an engineered glycosylation site at the N-terminus, positions 3–5) was translated in either the absence (lanes 1 and 2) or the presence (lanes 3 and 4) of microsomal membranes. In lanes 1 and 2, microsomal membranes were added posttranslationally (after 1 h, post-) and incubation was continued for another 1 h. Samples in lanes 1 and 3 were treated later with EndoH. The gels are representative of at least three independent experiments.

First, we performed *in vitro* E protein transcription/translation experiments in the presence of ER-derived microsomes and [^35^S]-labelled amino acids. The membrane insertion orientation of the predicted TM segment into microsomal membranes was based on N-linked glycosylation and summarized in figure 2*a*. N-linked glycosylation has been extensively used as topological reporter for more than two decades [8]. In eukaryotic cells, proteins can only be glycosylated in the lumen of the ER because the active site of oligosaccharyl transferase (OST), a translocon-associated protein responsible for N-glycosylation [9], is located there [10]; no N-linked glycosylation occurs within the membrane or in the cytosol. It is important to note that two possible N-linked glycosylation sites are located C-terminally of the predicted TM segment in E protein wild-type sequence at positions N48 and N66 (figure 1). However, N48 is not expected to be modified even if situated lumenally due to the close proximity of this glycosylation acceptor site to the membrane if the hydrophobic region is recognized as TM by the translocon [11,12]. Thus, mono-glycosylation (at N66) would serve as a C-terminal translocation reporter. To test N-terminal translocation, a construct was engineered where a predicted highly efficient glycosylation acceptor site (i.e. Asn-Ser-Thr, NST) was designed at the N-terminus. When E protein constructs were translated *in vitro* in the presence of microsomes, the protein was significantly glycosylated when the N-terminal designed glycosylation site was present, as shown by the increase in the electrophoretic mobility of the slower radioactive band after an endoglycosidase H (Endo H) treatment (figure 2*b*, lanes 1 and 2). However, when a control (Gln-Ser-Thr, QST) that is not a glycosylation acceptor site (lane 3) or the wild-type (lane 4) sequences were translated, E protein molecules were minimally glycosylated. Since multiple topologies have been reported for previous coronavirus E proteins [13–17], SARS-CoV-2 E protein insertion into the microsomal membranes in two opposite orientations cannot be discounted, but according to our data being dominant an Nt_lum_/Ct_cyt_ orientation.

### 2.3. E protein integrates cotranslationally into microsomal membranes

We have previously reported that several viral membrane proteins are cotranslationally inserted into ER-derived microsomal membranes [18–20]. Since membrane protein insertion and N-glycosylation are coupled at the ER by complex formation of a ribosome, the translocon and the OST [10], we sought to investigate whether or not SARS-CoV-2 E protein is cotranslationally inserted into the ER membrane by blocking protein synthesis after E protein (harbouring N-terminal NST glycosylation site) has been translated in the absence of membranes. As shown in figure 2*c*, E protein (NST) was efficiently glycosylated when microsomal membranes were added to the translation mixture cotranslationally (lane 4). But when microsomal membranes were included posttranslationally after the translation was inhibited by cycloheximide, the protein was only residually glycosylated (lane 2), suggesting that E protein is mainly integrated cotranslationally through the ER translocon. This means that the microsomal insertion machinery recognizes, orients and provides a path into the membrane for this viral protein.

### 2.4. Membrane topology in mammalian cells

**Figure 3.**
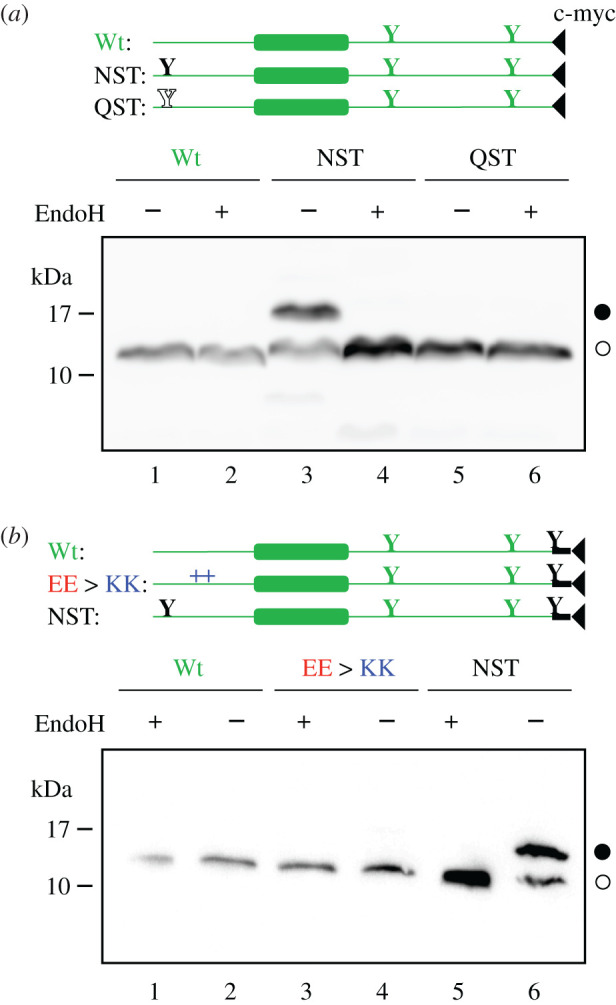
E protein topology in mammalian cells. To determine the topology *in vivo* HEK-293T cells were transfected with C-terminal tagged (c-myc) E protein variants. (*a*) Constructs encoding wild-type (Wt; lanes 1 and 2), inserted asparagine and threonine at positions 3 and 5 (NST; lanes 3 and 4) or glutamine and threonine at positions 3 and 5 (QST; lanes 5 and 6) were Endo H (+) or mock (−) treated. Filled and empty Y-shaped symbols denoted acceptor (NST) and non-acceptor (QST) glycosylation sites, respectively. (*b*) Additionally, we included constructs containing similar Wt (lanes 1 and 2), replaced glutamic acids at positions 7 and 8 by lysine residues (EE > KK; lanes 3 and 4) or NST (lanes 5 and 6) variants with an extra glycosylation site inserted at the Ct end of the protein. Once again, to confirm the glycosylated nature of the higher molecular weight bands, samples were either Endo H (+) or mock (−) treated. Designed glycosylation sites and tags are shown in black, while native E protein features are shown in grey.

To analyse protein topology in mammalian cells, a series of E protein variants tagged with c-myc epitope at the C-terminus were transfected into HEK-293T cells. As shown in figure 3*a*, only an E protein construct harbouring the N-terminal engineered acceptor site was efficiently modified (lanes 1–4), denoting an N-terminal ER luminal localization (Nt_lum_). Several topological parameters have been proposed to govern membrane protein topology, among which the preferential distribution of positively charged residues in the cytosol (positive-inside rule) has been established as the primary topology determinant both experimentally [21] and statistically [22]. E protein is a single-spanning membrane protein with an even net charge distribution on both sides of the membrane. There are only eight charged residues along the protein sequence (two negatively charged residues preceding the TM segment, and five positively and one negatively charged residues at the C-terminal domain; figure 1*a*), which correlates well the observed topology with the ‘positive-inside rule'. However, negatively charged residues have also been proved to significantly affect the topology [23]. To test the robustness of the observed topology, we added an optimized Ct glycosylation tag [24] and replaced the two negatively charged residues located in the translocated N-terminal domain (E7 and E8) by two lysine residues (figure 3*b*). In cells expressing this mutant E protein (EE > KK), the protein retained its C-terminal tail at the cytosolic side of the membrane as indicated by the absence of glycosylated forms (figure 3*b*, lanes 3 and 4). These data reveal that topological determinants have only a minor effect on viral membrane protein topology as previously demonstrated for other viruses [25] and suggest that viral membrane protein topology could have co-evolved with the protein environment of its natural host, ensuring proper membrane protein orientation. Altogether, the present *in vivo* results demonstrate that SARS-CoV-2 E protein is a single-spanning membrane protein with an Nt_lum_/Ct_cyt_ orientation in mammalian cell membranes. Similarly, SARS-CoV E protein was shown to mainly adopt an Nt_lum_/Ct_cyt_ topology in the infected cell and mammalian cells expressing the recombinant protein [26]. This topology is compatible with the ion channel capacity described previously [27], and with the recently published pentameric structural model of SARS-CoV E protein in micelles [28], in which the C-terminal tail of the protein is α-helical and extramembrane.

## 3. Concluding remarks

The membrane topology described here would allow the cytoplasmic C-terminal tail of the E protein to interact with the C-termini of M and/or S SARS-CoV-2 membrane-embedded proteins [3], and/or with Golgi scaffold proteins as previously described for other coronaviruses [29], to induce virus budding or influence vesicular traffic through the Golgi complex by collecting viral membrane proteins for assembly at Golgi membranes. Future experiments will have to unravel whether these functions involve the SARS-CoV-2 E protein.

## 4. Material and methods

### 4.1. Enzymes and chemicals

TNT T7 Quick for PCR DNA was from Promega (Madison, WI, USA). Dog pancreas ER rough microsomes were from tRNA Probes (College Station, TX, USA). EasyTag EXPRESS^35^S Protein Labeling Mix, [^35^S]-L-methionine and [^35^S]-L-cysteine, for *in vitro* labelling was purchased from Perkin Elmer (Waltham, MA, USA). Restriction enzymes were from New England Biolabs (Massachusetts, USA) and endoglycosidase H was from Roche Molecular Biochemicals (Basel, Switzerland). PCR and plasmid purification kits were from Thermo Fisher Scientific (Ulm, Germany). All oligonucleotides were purchased from Macrogen (Seoul, South Korea).

### 4.2. Computer-assisted analysis of E protein sequence

Prediction of transmembrane segments was done using up to 7 of the most common methods available on the Internet: ΔG Predictor [30,31] (http://dgpred.cbr.su.se/), TMHMM [32] (http://www.cbs.dtu.dk/services/TMHMM/), MEMSAT-SVM [33] (http://bioinf.cs.ucl.ac.uk/psipred/), TMpred (https://embnet.vital-it.ch/software/TMPRED_form.html), HMMTop [34] (http://www.enzim.hu/hmmtop/), Phobius [35] (http://phobius.sbc.su.se/) and TOPCONS [36] (http://topcons.net/). All user-adjustable parameters were left at their default values.

### 4.3. DNA manipulation

Full-length E protein was synthesized by Invitrogen (*GeneArt* gene synthesis) and subcloned into *Kpn*I linearized pCAGGS in-house version [37] using In-Fusion HD cloning Kit (Takara) according to the manufacturer's instructions. For *in vitro* assays, DNA was amplified by PCR adding the T7 promoter and the relevant glycosylation sites during the process. N-terminal NST glycosylation site was designed by inserting an asparagine and a threonine before and after Ser3, respectively. Control no-glycosylable QST site was introduced in similarly inserting a glutamine residue instead of an asparagine. All E protein variants were obtained by site-directed mutagenesis using QuikChange kit (Stratagene, La Jolla, California) and were confirmed by sequencing the plasmid DNA at Macrogen Company (Seoul, South Korea).

### 4.4. Translocon-mediated insertion into microsomal membranes

E protein variants, PCR amplified from pCAGGS, were transcribed and translated using the TNT T7 Quick for PCR DNA coupled transcription/translation system (Promega, USA). The reactions contained 10 µl of TNT, 2 µl of PCR product, 1 µl of EasyTag (5 µC_i_) and 0.6 µl of column-washed microsomes (tRNA Probes, USA) and were incubated for 60 min at 30°C. Translation products were ultracentrifuged (100 000*g* for 15 min) on a 0.5 M sucrose cushion and analysed by SDS-PAGE. For the endoglycosidase H (Endo H), the treatment was done as previously described [20]. Briefly, the translation mixture was diluted in 120 µl of PBS and centrifuged on a 0.5 M sucrose cushion (100 000*g* 15 min 4°C). The pellet was then suspended in 50 µl of sodium citrate buffer with 0.5% SDS and 1% β-mercaptoethanol, boiled 5 min, and incubated 1 h at 37°C with 1 unit of Endo H. Then, the samples were analysed by SDS-PAGE and gels were visualized on a Fuji FLA3000 phosphorimager using Image Reader 8.1j software.

### 4.5. Postranslational and cotranslational insertion assay

E protein DNAs were transcribed/translated (30°C 1 h) either in the absence (figure 2*c*, post- samples) or in the presence (co- samples) of microsomal membranes. The translation was inhibited with cycloheximide (10 min, 26°C, 2 mg ml^−1^ final concentration), after which microsomes were added to those samples labelled as posttranslational and incubated for an additional hour at 30°C. Subsequently, membranes were collected by ultracentrifugation; half of the samples were EndoH treated and analysed by SDS-PAGE (double volume was loaded for the post-samples due to the lower translation levels observed). Protein molecules were visualized on a Fuji FLA3000 phosphorimager.

### 4.6. E protein expression in mammalian cells

E protein sequence variants were tagged with a c-myc epitope at their C-terminus (Glu-Gln-Lys-Leu-Ile-Ser-Glu-Glu-Asp-Leu, EQKLISEEDL) and inserted in a pCAGGS-ampicillin plasmid. When appropriate (figure 3*b*), an optimized glycosylation site followed by a flexible dipeptide (Asn-Ser-Thr-Gly-Ser, NSTGS) [24,38] preceded the c-myc epitope. Once the sequence was verified, plasmids were transfected into HEK293-T cells using Lipofectamine 2000 (Life Technologies) according to the manufacturer's protocol. Approximately 24 h post-transfection cells were harvested and washed with PBS buffer. After short centrifugation (1000 r.p.m. for 5 min on a table-top centrifuge), cells were lysed by adding 100 µl of lysis buffer (30 mM Tris-HCl, 150 mM NaCl, 0.5% Nonidet P-40), sonicated in an ice bath in a bioruptor (Diagenode) during 10 min and were centrifugated. Total protein was quantified and equal amounts of protein submitted to Endo H treatment or mock-treated, followed by SDS-PAGE analysis and transferred into a PVDF transfer membrane (ThermoFisher Scientific). Protein glycosylation status was analysed by Western Blot using an anti-c-myc antibody (Sigma), anti-rabbit IgG-peroxidase conjugated (Sigma) and with ECL developing reagent (GE Healthcare). Chemiluminescence was visualized using an ImageQuantTM LAS 4000mini Biomolecular Imager (GE Healthcare).

## Data accessibility

This article does not contain any additional data.
